# Fatal Avian Influenza (H5N1) Infection in Human, China

**DOI:** 10.3201/eid1611.090212

**Published:** 2010-11

**Authors:** Ji Zhang, Xingyi Geng, Yanhui Ma, Shiman Ruan, Shuhui Xu, Lanzheng Liu, Huaru Xu, Guoliang Yang, Chunrong Wang, Chong Liu, Xiuyun Han, Qiuyan Yu, Hongqi Cheng, Zhan Li

**Affiliations:** Author affiliation: Jinan Municipal Center for Disease Control and Prevention, Jinan, People’s Republic of China

**Keywords:** Viruses, avian influenza, influenza-like illnesses, respiratory infections, poultry, H5N1, China, letter

**To the Editor:** Since the first avian influenza virus (H5N1) was isolated from a goose in the southern region of the People’s Republic of China a decade ago (*1*), no poultry outbreak has been reported in Shandong Province in eastern China, although adjacent provinces have experienced an avian influenza epidemic (*2*). In fall 2008, several rounds of investigation of poultry farms and markets were conducted in Jinan, Shandong Province, and no influenza virus (H5N1) was isolated by reverse transcription–PCR (RT-PCR) from 19,340 poultry oropharyngeal, cloacal, and cage specimens.

However, a fatal influenza (H5N1) infection in a human was identified on January 17, 2009 (*3*). The patient was a 27-year-old woman from Jinan. Influenza-like illness (ILI) developed on January 5, and the patient received intravenous ribavirin and cephalosporins on January 9. On January 11, she was hospitalized for fever (41°C) and respiratory symptoms. On January 15, extensive infiltration in both lungs developed; the diagnosis was pneumonia of unknown etiology. Early on January 17, she underwent endotracheal intubation. She died of acute respiratory distress syndrome and multiple organ failure later that day.

Two endotracheal aspirates collected on January 17 were positive for influenza virus (H5N1) and for genes encoding matrix protein by real-time PCR and RT-PCR. However, throat swabs collected on January 15 and 16 had been negative even after repeated testing (Table). The influenza virus (H5N1) was isolated on January 22 after 48-hour culture and named A/Shandong/1/2009(H5N1). Whole-genome sequencing showed that all segments were of avian origin. The hemagglutinin gene and amino acid sequences of this virus were highly homologous with 24 strains of influenza virus (H5N1) isolated during 2005–2008 in China. There was no change in the hemagglutinin cleavage or the receptor binding sites or in the neuraminidase gene conferring oseltamivir resistance (*4*). Nevertheless, mutations in the matrix 2 gene indicated amantadine resistance (*5*).

**Table:** Clinical specimens collected from patient with fatal avian influenza virus (H5N1) infection and test results, Shandong Province, China*

Specimen type	Collection date, Jan 2009	Place tested	Test method	Result
Nasopharyngeal swab	15	Shandong CDC	RT-PCR	A/H5HA/AN1 negative
Nasopharyngeal swab	15	Shandong CDC	Real-time PCR	A/H5HA/H5NA negative
Nasopharyngeal swab	16	Shandong CDC	RT-PCR	A/H5HA/AN1 negative
Nasopharyngeal swab	16	Shandong CDC	Real-time PCR	A/H5HA/H5NA negative
Endotracheal aspirate	17	Shandong CDC	RT-PCR	A/H5HA/AN1 positive
Endotracheal aspirate	17	Shandong CDC	Real-time PCR	A/H5HA/H5NA positive
Endotracheal aspirate	17	China CDC	RT-PCR	A/H5HA/AN1 positive
Endotracheal aspirate	17	China CDC	Real-time PCR	A/H5HA/H5NA positive

*CDC, Center for Disease Control and Prevention; RT-PCR, reverse transcription–PCR.

The patient was a stay-at-home mother with a daughter 20 months of age. Her husband operated a barbecue food stand. The raw poultry ingredients (duck blood and chicken hearts) for the barbecue were washed and processed at home, and the patient may have had unprotected contact with raw poultry products. However, the patient did not raise poultry, did not have contact with sick or dead poultry, had not visited a poultry market recently, and had not consumed sick or dead poultry or raw poultry food. No influenza virus (H5N1) was detected by RT-PCR from the raw duck blood and chicken hearts saved in the patient’s home refrigerator or at the poultry seller where the duck blood and chicken hearts originated. No influenza virus (H5N1) was detected by RT-PCR from 448 poultry oropharayngeal and cloacal specimens collected from the live poultry markets immediately after the virus was identified in the patient.

The patient’s close contacts (157 persons), including family members and healthcare professionals, were isolated and monitored medically for 7 days according to the Chinese Center for Disease Control and Prevention guidelines, but without chemoprophylaxis because oseltamivir was unavailable. ILI did not develop in any of these persons, and all had negative results for immunoglobulin M against influenza (H5N1) virus. During January 17–23, all 9,865 clinic or hospital visitors with respiratory symptoms in the patient’s residential region were interviewed; 829 had fever and 586 had ILI. All recovered quickly without treatment. Persons (537) with frequent exposure to poultry were monitored; ILI did not develop in any person.

This case was comparable to some other influenza (H5N1) infections in humans without identified sources of exposure (*6**,**7*). The virus for this case might have come from infected poultry products in the barbecue raw ingredients. Viremic blood from infected poultry can contaminate raw poultry products. It is also possible that blood was contaminated from poultry struggling during blood collection. The shared kitchen for cooking and raw poultry product processing might be the place where viral transmission occurred. Although the patient had no direct contact with raw poultry products, the shared utensils might have acted as vectors for transmission. Consequently, Jinan enhanced public education to increase awareness of personal protection for persons with direct contact with poultry or poultry raw products and their family members.

The failure to use oseltamivir resulted from lack of alertness and preparedness by healthcare professionals for influenza (H5N1) infection because no human influenza (H5N1) infection had been reported in Shandong Province. The lack of a local oseltamivir reserve also precluded timely oseltamivir use. In response to this public health incident, Jinan enhanced education on self-protection, case management, empirical oseltamivir use, and emergency response to influenza by healthcare and public health professionals, in addition to building a local oseltamivir reserve. These efforts led to preparedness and timely treatment with oseltamivir during the second case of influenza pandemic (H1N1) 2009 infection in China on May 11, 2009 (*8*).

Because influenza viruses (H5N1) can replicate efficiently only in cells of the lower respiratory tract where the avian virus receptor is prevalent (*9*), it is better to collect lower respiratory tract specimens early for laboratory testing. Given that early oseltamivir administration is critical and most effective (*10*), oseltamivir should be administered quickly to patients with pneumonia of unknown etiology without waiting for laboratory confirmation of influenza infection.
